# Children as controls in research; The voice of the volunteer

**DOI:** 10.1371/journal.pone.0325069

**Published:** 2025-06-03

**Authors:** Oskar W. Angenete, Janne Haustreis, Lisbeth K. Aune, Anette Lundestad, Lena Cetrelli, Thomas Angell Augdal, Ellen Berit Nordal, Kjersti Grønning, Marite Rygg

**Affiliations:** 1 Department of Radiology and Nuclear Medicine, St Olav Hospital HF, Trondheim University Hospital, Trondheim, Norway; 2 Department of Circulation and Medical Imaging, Faculty of Medicine and Health Sciences, Norwegian University of Science and Technology, Trondheim, Norway; 3 Department of Pediatrics, St. Olavs University Hospital, Trondheim, Norway; 4 Department of Pediatrics, University Hospital of Northern Norway, Tromsø, Norway; 5 Department of Clinical and Molecular Medicine, Faculty of Medicine and Health Sciences, Norwegian University of Science and Technology (NTNU), Trondheim, Norway; 6 Center of Oral Health Services and Research (TkMidt), Trondheim, Norway; 7 The Public Dental Health Service, Trøndelag County, Trondheim, Norway; 8 Section of Paediatric Radiology, University Hospital of North Norway, Tromsø, Norway; 9 Department of Clinical Medicine, Faculty of Health Sciences, UiT The Arctic University of Norway, Tromsø, Norway; 10 Department of Public Health and Nursing, Norwegian University of Science and Technology, Trondheim, Norway; 11 Department of Research, Nord-Trøndelag Hospital Trust, Levanger, Norway; Prince Sattam bin Abdulaziz University, SAUDI ARABIA

## Abstract

**Background:**

Modern paediatric healthcare is increasingly dependent on diagnostic methods such as imaging and blood samples. These methods require knowledge of physiology and anatomy to separate signs of early disease development from physiological variants. To acquire this knowledge, researchers depend on studies on children from the general populations, but little is known about how children and their guardians experience participation in studies. This study aims to map the experiences of children participating in research.

**Materials and methods:**

From a cohort of children and guardians from the general population, participating as controls in a large study on juvenile idiopathic arthritis (NorJIA), a subset was invited to share their experiences. The participants were examined using blood sampling, MRI, x-ray, bone-density scan, anthropometric measurements, and questionnaires. A questionnaire of 26 questions regarding their experiences from the study was sent out 2–4 weeks after the data collection.

**Results:**

The questionnaire was filled out by 50 children and 50 guardians. A large majority of the children responded that they were positive to help researchers, that blood sample procedures and imaging procedures went well, that they would participate in the study again, and would recommend their friends to take part in similar studies. Guardians generally responded positively, but there were diverging responses between children and guardians.

**Conclusion:**

Children from the general population reported mainly positive experiences from participation in research, including imaging and blood sampling. The experiences of the children and their guardians are sometimes diverging, underpinning the importance of addressing the child’s opinions.

## Introduction

Modern healthcare, including paediatric healthcare, has made great progress the last decades. A considerable part of the improvement can be attributed to better integration and development of laboratory and medical imaging methods in the clinical workflow. Increasing availability of advanced medical imaging enables clinicians across all specialties to diagnose and treat diseases at an earlier stage than before. The development of new laboratory methods represents tools for detection and categorization of diseases in an unprecedented way.

However, finding and characterizing signs of early disease development, especially in children, requires thorough knowledge of the physiology and anatomy and the ability to separate signs of early pathology from developmental and anatomical variations.

It is well known that reference values for several biochemical analytes differ widely in children from different age groups and adults. Newer biomarkers often lack standard references in children. As decision-making in modern healthcare often depends on laboratory results, the need for valid and relevant reference values is obvious [1,2].

The European Society of Paediatric Radiology states in their research agenda that documenting normal variants in imaging is a key aim, and as a promoter of research, they wish to facilitate further studies on this topic [3]. Even though the radiologic literature addressing development and anatomical variations is growing [4–8], there is still a lack of knowledge of the physiological appearance of the paediatric body with regards to imaging.

Conducting research on a paediatric population is ethically challenging. Children are often categorised as a vulnerable study population. Ethical guidelines propose that the risk associated with a study on a vulnerable population can only be accepted if there are no alternatives. Further, the studied group should stand to benefit from the results of the research, not only as a group, but also as individuals. In addition, the level of tolerable risk that can be accepted in a vulnerable population is specified as minimal [9]. However, the complicated interpretation of what can be seen as minimal risk might impose unwanted restrictions on research studies [10]. Risk assessment of research in medical imaging is complicated as it concerns both the risk of being informed on subclinical medical conditions and sometimes exposing the participant to radiation. On the other hand, ethical guidelines also recognize the need for children to participate in research. Children have the right to be treated with safe drugs, in terms of effect, tolerance, and pharmacokinetics. Children also have a right to safe and valid diagnostic procedures, derived from evidence-based medicine, as stated in the Declaration of Helsinki and by the Council for International Organizations of Medical Sciences [9,11].

Research agendas are rarely directly influenced by the opinions and experiences of children and young people. This is a topic that is seldom problematized [12]. The fact that the impact of the direct opinion of the children (the «user perspective”) probably is under-communicated, could lead to suboptimal study design [12]. Generally, acknowledging the user’s perspective and experiences in research is encouraged by both research funders and the research community. Further studies on how the user perspective affects study protocol and outcomes are encouraged [13]. There seems to be a lack of knowledge on how children experience participating in research as controls, especially focusing on imaging and reference values of blood samples. Hence, addressing the user perspective, also in research involving controls, is important.

The present study aims to map the motivation and experiences of young volunteers from the general population and their guardians participating in research. Hopefully, the results can be used by institutional review boards (IRB) and researchers as an empirical database for future research with similar ethical considerations.

## Materials and methods

### Study design

The NorJIA study is a Norwegian longitudinal prospective multicentre study on juvenile idiopathic arthritis (JIA) (Clinicaltrials.gov identifier NCT03904459) with study start 2015-03-25 and study completion 2020-06-20. The participants were children aged 4–16 years from the Western, Central, and Northern Health Region of Norway, with a JIA diagnosis according to the International League of Associations for Rheumatology (ILAR) [14]. As part of the NorJIA study, age- and gender-matched controls from the general population without a chronic inflammatory disease, were invited to participate in the study. The eligible controls were randomly chosen among children receiving free, oral health examinations as part of the Norwegian dental health service program in the Central, Western, and Northern Health Region of Norway. The response rate for the controls was 76% (224/294). The children were assessed twice, two years apart. The participants were offered 300 NOK (24 euro) as compensation for participation in addition to travel expenses.

In the present study, a cohort from the control group of the second visit in the NorJIA study was invited to participate in a mixed model study including both quantitative and qualitative methods. The aim of the study required an amendment to the original NorJIA research protocol, and consequently, a separate approval from the research ethics committee was required. Therefore, due to time constraints, only part of the NorJIA control group and only from the Central and Northern Health Region of Norway, were eligible for inclusion. The quantitative part of this study is presented here.

### Inclusion

Inclusion criteria was participation in the NorJIA study as a control with informed consent for this part of the study. During their second visit in the NorJIA study, the controls were approached by a research nurse and asked if they wanted to participate in the present sub-study. It was clearly pointed out that this was an additional study, and that participation was voluntary and independent of the participation in other parts of the NorJIA study. The oral and written information included a short presentation of the background for the study, what data would be collected, possible advantages and disadvantages with participation, data handling, and an explanation on how to withdraw from the study. All participants were invited together with their parents or legal guardians (from here on referred to as guardians).

### Data collection

Participants who accepted the invitation were examined with a standardized set of examinations including anthropometric measurements (height and weight), x-ray radiography of the left hand, dual energy x-ray absorptiometry scan (DXA), blood samples, questionnaires on physical activity, diet, and pubertal development. Children could choose whether they wanted to participate in all, or only selected examinations. Due to lack of resources and availability of magnetic resonance imaging (MRI), only a small proportion of the study group was offered an unenhanced MRI of the temporomandibular joints. During the day of the examinations, which usually lasted 3–4 hours, the participants and their guardians were accompanied by a study nurse who facilitated access to the different parts of the hospital, performed the clinical examinations, and helped with access to the digital questionnaires, when needed.

### Study questionnaire

A questionnaire from the Norwegian Institute of Public Health was used as a framework. The questionnaire was originally designed and validated to measure paediatric patient experiences in outpatient diabetes care and the questions were therefor adapted to suit the present study. Further, the questionnaire was in Norwegian and made available for free by the Norwegian Institute for Public Health, making it suitable for the present study in terms of resources and availability. The questionnaires were sent to the participants by mail 2–4 weeks after the day of the examinations and were returned by mail. The questionnaire was translated to English for the purpose of this publication. The questionnaire consisted of 25 questions, exploring the participant’s experiences before, during, and after the day of the examinations. The questionnaire was divided in two similar parts, one for the child and one for the guardian. Children <12 years of age were allowed assistance from their guardian during completion of the questionnaire. The questionnaires were filled out at home and the interaction between the child and the guardian was therefore not possible to analyse.

### Statistics

A five-grade (1–5) Likert score was used as response to each question, ranging from “Not at all”, “to a small extent”, “to some extent”, “to a large extent” or “to a very large extent”. We used proportions to describe the answers of the participants. To define a positive response, a cutoff between “to some extent” and “to a large extent” was chosen. Cohen’s weighted Kappa was used to determine the agreement between the answers of the child and the guardian. Kappa score below 0 was considered poor, agreement ranging from 0–0.2 was considered slight, 0.21–0.4 fair, 0.41–0.6 moderate, 0.61–0.8 substantial, and 0.81–1 almost perfect [15]. IBM SPSS Statistics version 29 was used for statistical calculations.

### Ethics

The Regional Committees for Medical and Health Research Ethics of Western Norway (REK-Vest) approved the study (REK 2012/542). Children were informed orally and in written text according to their age. Written consent was obtained from all participants. For children under 16 years, legal guardians approved the study on behalf of their children.

## Results

In the cohort eligible for inclusion, 107 of 207 children were invited to participate together with their guardians. The questionnaires were filled out and returned by 50 children (33 girls). Demographic characteristics are displayed in Table 1. Compared to the full NorJIA control group, the level of parental education tended to be higher in the present study. One child did not answer all the questions. Among the guardians, 50 returned the questionnaires of which 39 were filled out by the mother, nine by the father, and two questionnaires filled out by both the mother and the father. Most children participated in all examinations. One child did not participate in the DXA scan, four children did not undergo x-ray of the left hand, four children did not participate in blood sampling. As MRI resources were scarce only 10 participants were offered and underwent MRI.

**Table 1:** Characteristics of 50 participating children and guardians compared to the control group of the NorJIA study.

	Respondents	NorJIA control group
Age, child, median (IQR)	13 (10–16)	12 (10–15)
Girls, n (%)	33 (67%)	124 (60%)
**Parental education** ^*^		
*Primary school (≤10 years), n (%)*	0	2 (1)
*High school (11–13 years), n (%)*	2 (7)	32 (23)
*University ≤ 4 years, n (%)*	15 (50)	39 (28)
*University > 4 years, n (%)*	13 (43)	46 (33)

* Defined by the guardian with the highest education. Missing data for 20 respondents and 68 from the control group.

### The children’s opinion

**Fig 1:**
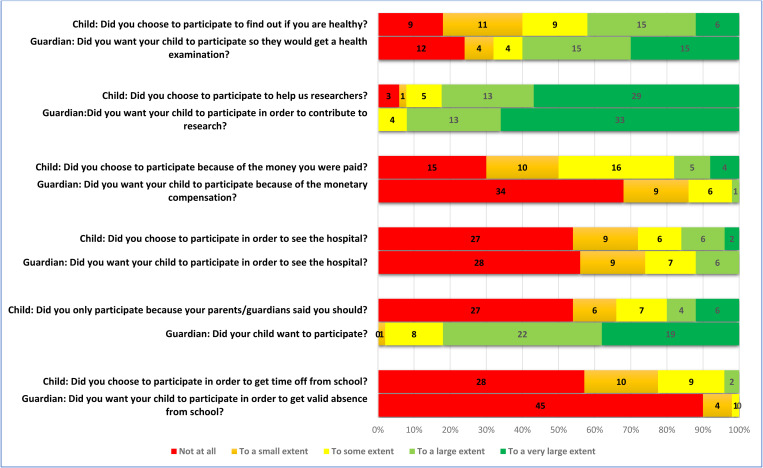
Responses of the children (n = 50) and their guardians (n = 50) addressing their motivation for participation.

**Fig 2:**
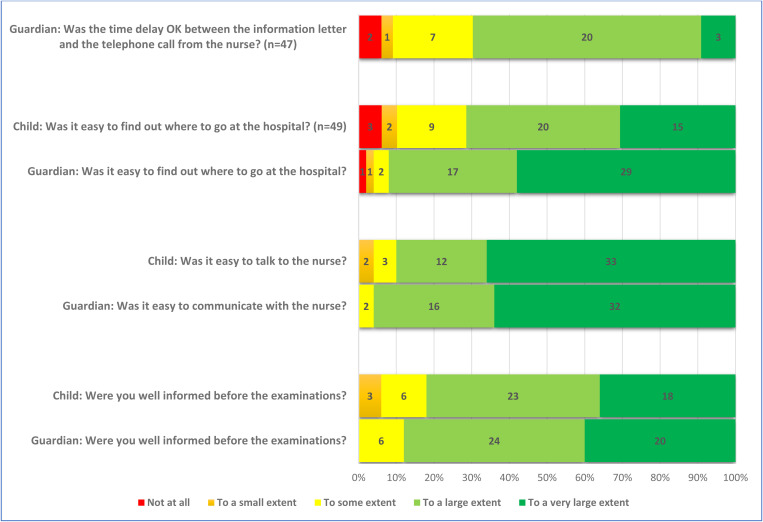
Responses of the children (n = 50) and their guardians (n = 50) unless specified, addressing information to the participants before the examinations.

**Fig 3:**
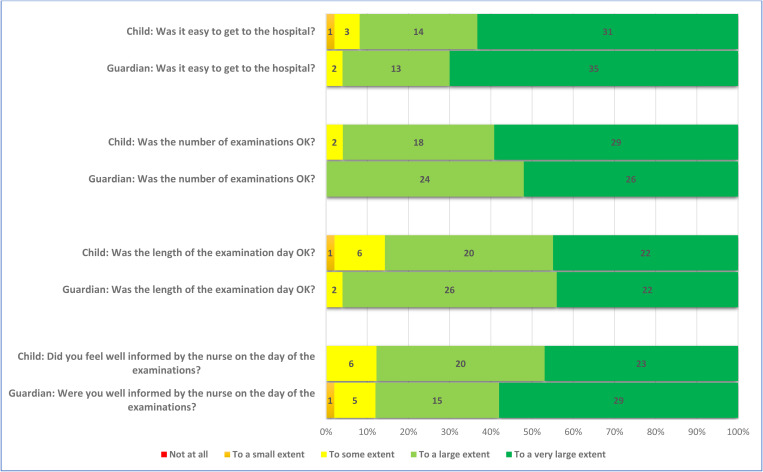
Responses of the children (n = 49) and their guardians (n = 50) addressing their experience of the day at the hospital.

**Fig 4:**
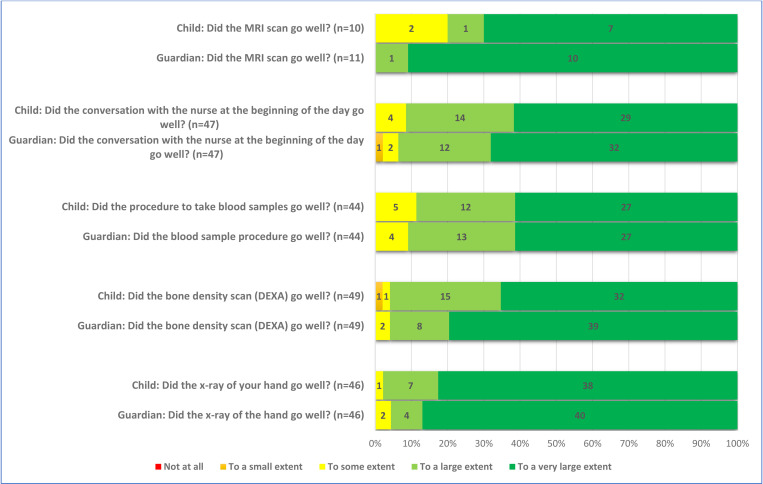
Responses of the children and their guardians addressing their experience of the different examinations.

**Fig 5:**
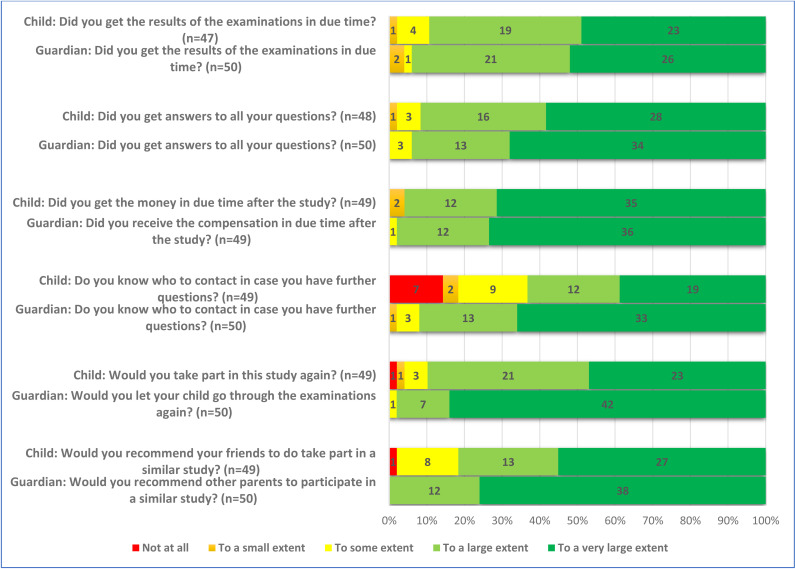
Responses of the children and their guardians addressing their thoughts after the study.

A large majority of the children (82%, n = 41/50) were positive to participate in order to help researchers, and 18% (9/50) responded that they participated because of the money they received. Further, 6% (3/50) responded that they participated to have a day off from school (Figs 1 and 2). Most children felt well informed before the examinations (82%, n = 41/50) (Fig 2). The respondents also conveyed that they had positive experiences related to communication with the nurse at the examinations, 88% (43/49) (Fig 3). Nearly all stated that the DXA scan and the x-ray went well, 96% (47/49) and 98% (45/46), respectively. Positive responses were received by 80% (8/10) of the children who underwent MRI scan. The response “to some extent” was received from 20% (2/10) of the children. Again, a majority of the children, 89% (39/44), responded that the blood sample procedure went well (Fig 4). When asked if they would go through the study again, nearly all, 94% (46/49), responded positively. Finally, 80% (40/49) of the children responded positively to the question “Would you recommend your friends to do a similar study?” (Fig 5). The responding children were grouped according to age < 12 years and ≥ 12 years. Subgroup analyses according to age groups were performed on question 2, 17, 18, and 24 without larger differences between the groups. Regarding question 24; “Would you take part in this study again?” there was a trend towards more positive responses in the older age group. The low number of participants did not allow for calculation of statistical significance.

### The guardians’ opinion

A large majority of the guardians, 92% (46/50), were also positive to participate in order to help researchers. The monetary compensation for the child was less important for the guardians than for the children, 2% (1/50). Nearly all the guardians were positive to letting their child go through the study again, 98% (49/50). All the guardians, 100% (50/50), would recommend other parents to let their child participate in a similar study (Fig 5).

### Comparing children’s and guardians’ opinion

Although both children and guardians generally were positive, the response of the individual child and the individual guardian was not necessarily the same. The agreement between the children and their guardians varied widely, assessed by Cohen’s weighted kappa. The respondents showed slight agreement on the question “Did you wish to participate to help us researchers?” and fair agreement on the question “Did you wish to participate because of the gift card?” (Table 2). On the latter question, only one guardian, but nine children responded positively. There was also fair agreement on the question “Would you do this study again?”, where two children were negative to doing it again, but none of the guardians. There was also fair agreement on the question “Would you recommend your friends to do a similar study?” where none of the guardians were negative, but one child would not recommend participation.

**Table 2:** Agreement between the children’s and guardians’ responses measured with Cohen´s weighted Kappa.

Questions	Kappa (95% CI)
C: Did you choose to participate to find out if you are healthy? G: Did you want your child to participate so they would get a health examination?	0.6 (0.41-0.79)
C: Did you choose to participate to help us researchers? G: Did you want your child to participate to contribute to research?	0.07 (−0.15-0.28)
C: Did you choose to participate because of the money you were paid? G: Did you want your child to participate because of the monetary compensation?	0.25 (0.04-0.46)
C: Did you choose to participate in order to see the hospital? G: Did you want your child to participate in order to see the hospital?	0.44 (0.16-0.72)
C: Did you only participate because your parents said you should? G: Did your child wish to participate?	N/A*
C: Did you choose to participate in order to get time off from school? G: Did you want your child to participate to get valid absence from school?	0.17 (−0.01-0.35
C: Was it easy to find out where to go at the hospital? G: Was it easy to find out where to go at the hospital?	0.33 (−0.04-0.70)
C: Was it easy to talk to the nurse? G: Was it easy to communicate with the nurse?	0.18 (0.21-0.78)
C: Were you well informed before the examinations? G: Were you well informed before the examinations?	0.50 (0.21-0.78)
C: Was it easy to get to the hospital? G: Was it easy to get to the hospital?	0.25 (−0.06-0.56)
C: Was the number of examinations OK? G: Was the number of examinations OK?	0.17 (−0.11-0.44)
C: Was the length of the examination day OK? G: Was the length of the examination day OK?	0.23 (−0.08-0.54)
C: Did you feel well informed by the nurse on the day of the examinations? G: Were you well informed by the nurse on the day of the examinations?	0.39 (0.20-0.57)
C: Did the MRI scan go well? (n = 10) G: Did the MRI scan go well? (n = 11)	N/A**
C: Did the conversation with the nurse at the beginning of the day go well? G: Did the conversation with the nurse at the beginning of the day go well?	0.32 (0.11-0.53)
C: Did the procedure to take blood samples go well? G: Did the blood sampling procedure go well?	0.47 (0.16-0.79)
C: Did the bone density scan (DEXA) go well? G: Did the bone density scan (DEXA) go well?	0.69 (0.46-0.92)
C: Did the x-ray of your hand go well? G: Did the x-ray of the hand go well?	0.32 (−0.04-0.68)
C: Did you get the results of the examinations in due time? G: Did you get the results of the examinations in due time?	0.30 (−0.13-0.72)
C: Did you get answers to all your questions? G: Did you get answers to all your questions?	0.00 (−0.34-0.33)
C: Did you get the money in due time after the study? G: Did you receive the compensation in due time after the study?	0.24 (−0.05-0.52)
C: Do you know who to contact in case you have further questions? G: Do you know who to contact in case you have further questions?	0.16 (−0.06-0.38)
C: Would you take part in this study again? G: Would you let your child go through the examinations again?	0.31 (0.00-0.63)
C: Would you recommend your friends to take part in a similar study? G: Would you recommend other parents to participate in a similar study?	0.22 (0.03-0.40)

C = Child, G = Guardian, *Q5 is reversed from child to guardian, making kappa calculation inadequate. Q7 was only for guardians, ** Too low n for kappa calculation

## Discussion

In a population-based cohort of 50 children and 50 guardians who participated as controls in the NorJIA study, we have shown that a large proportion of both the children and their guardians had positive experiences during the data collection. Self-centered goals, such as monetary reward and getting a day off school, were not dominating factors for accepting to participate in the study. Instead, 82% of the children agreed that they participated to help researchers. Most of the respondents felt it was OK to participate in imaging and even in blood test sampling. A large majority of both the children and their guardians responded that they would do the study again and that they would recommend their peers to participate in a similar study.

Considering statements and publications from both National Research Ethics Committees and social science [16,17], it is important to involve children and to address their concerns regarding participation in research. Further, research on a paediatric and adolescent population is encouraged by both healthcare providers and regulators of healthcare [3,9,11]. In this perspective, our results constitute a piece of knowledge contributing to involvement of children in research.

Our results are in line with the results of Wendler [18], who found that most of their participants were proud of contributing to research for the benefit of others, and not themselves. Their respondents were also willing to accept risks in research if it could help other children/adolescents. The present study and the Wendler study differ in terms of the participants, where the Wendler study included 80% patients with a broad variety of diseases, and only 20% of the respondents were considered healthy.

A qualitative study was performed in 2015 on 25 young people, both patients and healthy individuals, between 10 and 25 years [19]. The concept of “helping others” recurred in many transcripts, where “others” was found to denote future patients, parents of patients, doctors, or more abstractly, a moral duty and an acknowledgment of the contribution of past generations. We would argue that there are probably similarities in terms of altruism in the responses in both the present study and the qualitative study, even though the studies obviously differ widely in terms of methodology.

In 2022, a study on the perceived experiences of paediatric patients (cancer survivors and very preterm children) and healthy controls during research MRI scans was published [20]. A majority of the subjects in the study experienced no or almost no fear and discomfort. These are reassuring results, similar to the results from the present study, but the differences in the included cohort should be noted, as results from patients not necessarily are transferrable to volunteering children from the general population.

A similar study was performed in the Netherlands [21]. The authors present results on 357 children drawn from four research studies where the children expressed their level of discomfort in certain medical procedures, including MRI and venepuncture. Most participants experienced discomfort comparable to a dentist check-up during MRI and venepuncture, which could be interpreted as slightly worse than the results from the present study. The included patient population was categorised as “clinical research”. It is therefore unclear whether the participants were volunteering into research or if they were included during their clinical work-up.

Ethical research guidelines state that both children and adults have the right to be informed about the potential risks and benefits of participation in research. An important publication from rural Uganda emphasizes that information to the potential participants before enrolment in research must be seen in a cultural and historical context, recognizing and acknowledging the concerns that participants might have before accepting to take part in research [22]. In our study, we have shown that the children and their guardians felt well informed and taken care of before, during, and after the study. Thereby, their potential concerns and questions hopefully were adequately addressed.

In the publication on ethical guidelines [9], CIOMS state that no more than minimal risk can be tolerated in children who do not stand to benefit from their participation in research. Risk assessment is often difficult and subjective. However, we do know that unenhanced MRI is not associated with physical risk and that the radiation dose of a single x-ray of the hand is very low (0.06 mSv) which is close to negligible [23]. Taken together with the results of the present study, one could argue that imaging research in similar settings would not constitute more than a minimal risk for the participant.

Today, when including patients and control volunteers in studies, the research community acknowledge the need to inform both paediatric and adult participants in research about the study, associated risks, the participant’s rights to withdraw, and data management. There is also widespread acceptance for the need to specifically inform the child, not only it’s guardians. We have shown that there is often poor or fair agreement between the responses from the child and the guardian. Some of the calculations of kappa were hampered by skewed distribution of responses, a known drawback with Cohen’s method of measuring agreement. To the best of our knowledge, this lack of agreement has not been published before. The results indicate the need to see the paediatric research participant as a decision-maker with equal right to be heard as guardians.

We have shown that most control participants experienced the data collection as positive and that they would do it again, if asked to. As mentioned earlier, research on children can more easily be justified if the individual stands to benefit from his or her participation. In their qualitative study from 2017, Staphorst argues that healthy children participating in research experience a real, individual benefit in terms of learning more about themselves, learning more about the healthcare system, having fun during the procedures, and, not least, experiencing the altruistic aspect of participating in research [24]. Together with the arguments from Staphorst, our results indicate that children from the general population participating in research as controls experience an individual benefit from their participation. From the IRB perspective, this could possibly be seen as a factor tipping the scales in favour of approval of research studies involving presumably healthy children.

The results of the present study can be used by IRBs to better understand both the positive and negative effects of participating in research as a child. The results can hopefully also be used by other researchers in paediatric healthcare as an indication on how a control group could experience their study. Potentially, the results could also be used in an educational setting where healthcare staff inform patients and guardians about the presumed experiences of a planned medical examination.

The strength of this study is that the study cohort consists of children and guardians from the general, paediatric population, not patients or controls with a specific condition. This makes the results more easily transferrable to children in the general population.

There are inherent limitations in quantifying the experiences of human beings, both adults and children. A quantitative method such as a Likert score can only to a certain extent explain how and what an individual felt and experienced. As such, the results of the study must be seen only as a part of the jigsaw puzzle that tries to picture human feelings and experiences. Another potential limitation is that all the participants in this study had already accepted participation in the NorJIA study and may thereby have been more inclined to positive responses. Further, one cannot exclude the possibility that the respondents were biased by social desirability, making them more inclined to respond positively. The study was carried out by mailing the participants the questionnaires. A certain degree of recall bias can therefore be expected as the participants could not fill out the questionnaires immediately after the examinations. However, the recall period was short and similar for all participants, and we wanted to give the participants some time to reflect on their experiences before they answered the questionnaire. We used Cohen’s Kappa to measure disagreement between the children’s and the guardians’ answers. In skewed datasets as in parts of the present study, Cohen’s Kappa produces low values, which is a known limitation of the method. However, we chose to use Cohen’s Kappa because it is the advised method for categorical variables and because other methods would be associated with larger limitations. As the participants were drawn from the general population, the results cannot be transferred to a population of patients with a known disease. Lastly, an important limitation is that the questionnaire does not address the perceived risk of participating in research, neither for the child, nor the guardian. A risk-benefit assessment could constitute an important factor when choosing to participate in research or not.

In the future, the authors would suggest studies on the qualitative aspects of participation in research. Further, larger studies addressing the differences between younger and older children and adolescents would be important contributions to the knowledge on children participating in research.

## Conclusion

In this study on children from the general population participating in research we found that a large proportion of the participants have positive experiences from their participation and would do the study again if asked to. Both blood sampling procedures and imaging procedures were associated with positive experiences. However, the experience of the child is not always in agreement with the experience of the guardian, underpinning the importance of addressing the child’s opinions.
